# Improvement of Broad-Spectrum Disease-Resistant Rice by the Overexpression of *BSR1* via a Moderate-Strength Constitutive Promoter and a Pathogen-Inducible Promoter

**DOI:** 10.3390/plants13081138

**Published:** 2024-04-18

**Authors:** Satoru Maeda, Shingo Goto, Haruhiko Inoue, Haruka Suwazono, Hiroshi Takatsuji, Masaki Mori

**Affiliations:** 1 Institute of Agrobiological Sciences, National Agriculture and Food Research Organization, Tsukuba 305-8634, Japan; gotos@affrc.go.jp (S.G.); haruhiko@affrc.go.jp (H.I.); morimasa@affrc.go.jp (M.M.); 2 Institute of Fruit Tree and Tea Science, National Agriculture and Food Research Organization, Shizuoka 424-0292, Japan; 3 Department of Applied Biological Science, Tokyo University of Science, Noda 278-8510, Japan

**Keywords:** BSR1, rice, RLCK, disease resistance, *Pyricularia oryzae*, *Xanthomonas oryzae* pv. *oryzae*, *Cochliobolus miyabeanus*, *Burkholderia glumae*

## Abstract

Conferring crops with resistance to multiple diseases is crucial for stable food production. Genetic engineering is an effective means of achieving this. The rice receptor-like cytoplasmic kinase BSR1 mediates microbe-associated molecular pattern-induced immunity. In our previous study, we demonstrated that rice lines overexpressing *BSR1* under the control of the maize ubiquitin promoter exhibited broad-spectrum resistance to rice blast, brown spot, leaf blight, and bacterial seedling rot. However, unfavorable phenotypes were observed, such as a decreased seed germination rate and a partial darkening of husked rice. Herein, we present a strategy to address these unfavorable phenotypes using an *OsUbi7* constitutive promoter with moderate expression levels and a pathogen-inducible *PR1b* promoter. Rice lines expressing *BSR1* under the influence of both promoters maintained broad-spectrum disease resistance. The seed germination rate and coloration of husked rice were similar to those of the wild-type rice.

## 1. Introduction

Stable crop production is an important agricultural issue because some countries are facing global food shortages owing to population growth, deteriorating security, and global warming. Rice is one of the most important crops worldwide and serves as a staple food for approximately 50% of the world’s population [1]. However, stable rice production is limited by diseases. Approximately 10–30% of the harvest is lost due to blast, one of the seven major crop diseases in the world, caused by the fungus *Pyricularia oryzae* in rice. However, 10% of the harvest is sufficient to feed 60 million people annually [2]. The bacterial leaf blight caused by *Xanthomonas oryzae* pv. *oryzae* (*Xoo*) is an important rice disease that has caused significant yield losses in Southeast Asian and West African countries [3]. Minimizing these losses would contribute to stabilizing and increasing rice production.

Addressing this problem and enhancing host resistance to these pathogens without relying on pesticides is the most economical and environmentally friendly approach to sustainable food production. Breeding a rice variety with broad-spectrum resistance (BSR) is the safest and most efficient method to achieve this goal. To date, *R* gene introduction has been the main method used for breeding disease-resistant varieties of rice [4]. However, in many cases, *R* gene-introduced varieties have only been effective in agricultural production for a few years because of the emergence of new pathogen biotypes that can overcome this gene [5]. In addition, the *R* gene generally confers resistance to a specific race of the pathogen but does not confer BSR. Furthermore, pyramiding these *R* genes using conventional breeding methods is labor-intensive, time-consuming, and difficult because of the need to remove linked unfavorable traits. Therefore, we considered using BSR genes as a feasible approach.

To date, several BSR genes have been identified. BSR is classified into species-non-specific (SNS) BSR, which confers resistance against two or more pathogen species, and race-non-specific (RNS) BSR, which confers resistance against two or more races or strains of the same pathogen [6]. SNS *BSR* is more valuable in agriculture than RNS BSR. Examples of SNS BSR genes include *WRKY45* [7] and *OsSSI2* [8], which are involved in providing disease resistance to *P. oryzae* and *Xoo* via the salicylic acid pathway. *WRKY30* [9] and *OsACS2* [10] are involved in providing disease resistance to *P. oryzae* and the sheath blight fungus *Rhizoctonia solani* through the biosynthesis of jasmonic acid and ethylene, respectively.

*BSR1* is an SNS BSR gene, and the BSR1 protein (OsRLCK278) is classified in the RLCK-VII protein family as BIK1 [11], PBL19, and PBL20 [12], which are well known to encode receptor-like cytoplasmic kinases (RLCKs) involved in providing disease resistance in *Arabidopsis* [13]. We have previously reported the disease resistance mechanism of *BSR1* [14,15,16,17].

The *BSR1* gene, when highly expressed under the maize ubiquitin promoter (P*_ZmUbi_*), confers SNS BSR. We have reported that *BSR1*-overexpressing (OX) rice shows strong resistance to *Xoo* and *Burkholderia glumae*, causing bacterial seedling rot, grain rot, *P. oryzae*, and brown spot fungus *Cochliobolus miyabeanus* [13,18]. There are few resistance genes for *B. glumae* and *C. miyabeanus*, although global warming is expected to increase their propagation in the future [19]. No other genes showed resistance to four or more of these diseases, making the use of *BSR1* in breeding desirable. However, because P*_ZmUbi_*-*BSR1* rice plants express high levels of *BSR1* throughout the plant body, unfavorable phenotypes, such as reduced seed germination rate and partial darkening of husked rice, have been observed [13,18]. Thus, we used a constitutive promoter with weaker activity than P*_ZmUbi_* and a pathogen-inducible promoter to generate rice lines expressing *BSR1* and evaluated their disease resistance, germination rate, and brown rice color.

## 2. Results

### 2.1. Enhancement of BSR1 Expression in Rice and Screening for Bacterial Leaf Blight Resistance

**Figure 1:**
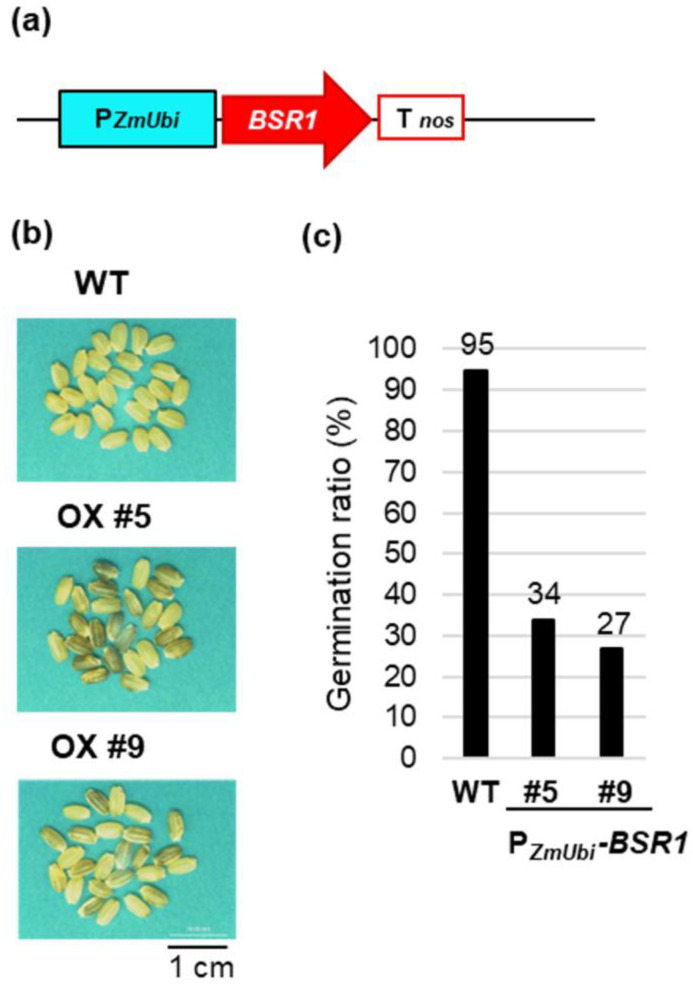
Undesirable phenotype of *BSR1* overexpression by maize ubiquitin promoter in rice seeds: (**a**) Maize ubiquitin promoter (P*_ZmUbi_*)-*BSR*1 construct. T*_nos_*, *nos* terminator. (**b**) Grain color of wild-type (WT) P*_ZmUbi_*-*BSR1* (OX) #5 and #9 lines. (**c**) Germination ratio (%) of WT P*_ZmUbi_*-*BSR1* #5 and #9 lines. *n* = 116–198.

We previously reported rice plants overexpressing *BSR1* using a construct (Figure 1a) in which *BSR1* cDNA was inserted downstream of the maize ubiquitin promoter (P*_ZmUbi_*), which is a constitutively high-expressing promoter [13]. P*_ZmUbi_*-*BSR1* transgenic rice lines #5 and #9 exhibited 159- and 130-fold higher expression of *BSR1* than the wild-type (WT), respectively, and demonstrated BSR to at least the four pathogens described above [13,18]. However, regarding growth and morphological characteristics, the P*_ZmUbi_*-*BSR1* lines produced partially blackish brown rice (Figure 1b), and the seed germination rate was reduced to approximately 1/3 to 1/4 of the original (Figure 1c), whereas the brown rice color in the WT was white (Figure 1b). We attributed this undesirable trait to the use of a strong promoter and considered that this problem could be resolved by improving the promoter. Therefore, we expressed *BSR1* using the rice ubiquitin promoter (P*_OsUbi7_*) [20], a constitutive promoter with weaker activity than P*_ZmUbi_*, and the *OsPR1b* promoter (P*_PR1b_*) [21], whose activity was induced by infection with *P. oryzae* and *Xoo.*

**Figure 2:**
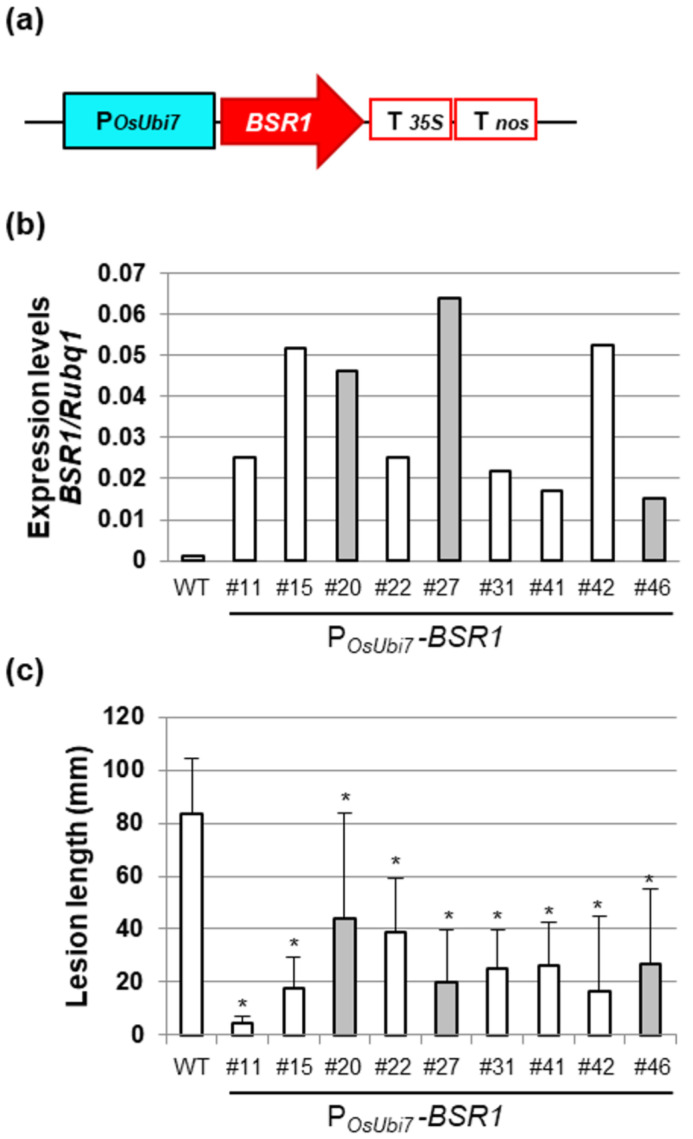
Generation and screening of *P_OsUBi7_*-*BSR1* lines: (**a**) Rice *Ubi7* promoter (P*_OsUbi7_*)-*BSR1* construct. T*_35S_*, CaMV*35S* terminator; T*_nos_*, *nos* terminator. (**b**) *BSR1* expression levels in P*_OsUbi7_*-*BSR1* T0 lines. (**c**) Disease resistance to *Xanthomonas oryzae* pv. *oryzae* (T7174, race I) in P*_OsUbi7_*-*BSR1* T1 lines. Lesion lengths in P*_OsUbi7_*-*BSR1* plants were significantly lower than those in WT plants (* *p* < 0.05 by Dunnett’s test). Values are mean ± standard deviation (*n* = 4–14). Gray bars in (**b**,**c**) indicate that blackish grains were included, whereas white bars indicate that only white grains were included.

P*_OsUbi7_*-*BSR1* rice lines were generated using a vector in which *BSR1* cDNA was inserted between the P*_OsUbi7_* promoter and double terminator (Figure 2a). From approximately 50 T0 plants carrying P*_OsUbi7_*-*BSR1*, lines resistant to *Xoo* race 1 were selected through initial screening, and the expression levels of *BSR1* were subsequently analyzed. Next, we verified the resistance of the selected lines in T1 plants and selected nine lines that demonstrated approximately half or less suppression of lesion length compared with the WT. The expression levels of *BSR1* and resistance levels to *Xoo* in the nine selected lines are presented in Figure 2b,c, respectively. Figure S1 presents *Xoo* resistance in three representative lines. Additionally, since the P*_ZmUbi_*-*BSR1* rice is also resistant to other *Xoo* races [18], we inoculated race III *Xoo* to lines #15 and #22 as representatives. Therefore, the lesion lengths were reduced to <1/4 of that in the WT, indicating resistance (Figure S2). Then, the grain color of the T1 seeds from these lines was examined. Three lines (#20, #27, and #46) showed blackish grains (Figure 2b,c, gray bar); however, six lines (#11, #15, #22, #31, #41, and #42) exhibited white grains similar to those of the WT (Figure 2b,c, white bar). The P*_OsUbi7_*-*BSR1* lines showing *Xoo*-resistant and white grains (Figure 2b, white bar only) had 16.0- to 49.5-fold higher expression of *BSR1* than that of WT, which was lower than those of the P*_ZmUbi_*-*BSR1* rice described above. We successfully generated lines with leaf blight resistance and white grains using the *OsUbi7* promoter.

**Figure 3:**
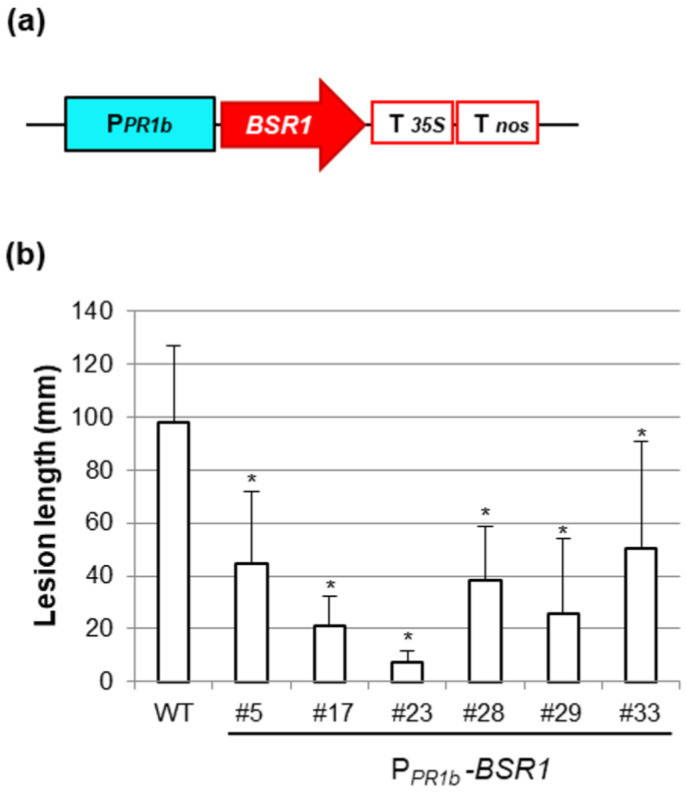
Generation and screening of P*_OsPR1b_*-*BSR1* lines: (**a**) Rice *PR1b* promoter (P*_PR1b_*)-*BSR1* construct. T*_35S_*, CaMV*35S* terminator; T*_nos_*, *nos* terminator. (**b**) Disease resistance to *Xanthomonas oryzae* pv. *oryzae* (T7174) in P*_PR1b_*-*BSR1* T1 lines. Lesion lengths in P*_PR1b_*-*BSR1* plants were significantly lower than those in WT plants (* *p* < 0.05 by Dunnett’s test). Values are mean ± standard deviation (*n* = 3–9). White bars indicate that only white grains were included.

P*_PR1b_*-*BSR1* rice lines were generated using a vector in which *BSR1* cDNA was inserted between the P*_PR1b_* promoter and the double terminator, as illustrated in Figure 3a. From approximately 30 T0 plants carrying P*_PR1b_*-*BSR1*, lines resistant to *Xoo* race 1 were selected through initial screening. By confirming the resistance of the selected lines in T1 plants, six lines that exhibited approximately half or less suppression of lesion length compared with the WT were identified (Figure 3b). The grain color of the T1 seeds from these lines was then examined, and all six lines showed a white grain color (Figure 3b, white bar) similar to that of the WT.

### 2.2. Other Disease Resistance in P_OsUbi7_-BSR1 Lines

**Figure 4:**
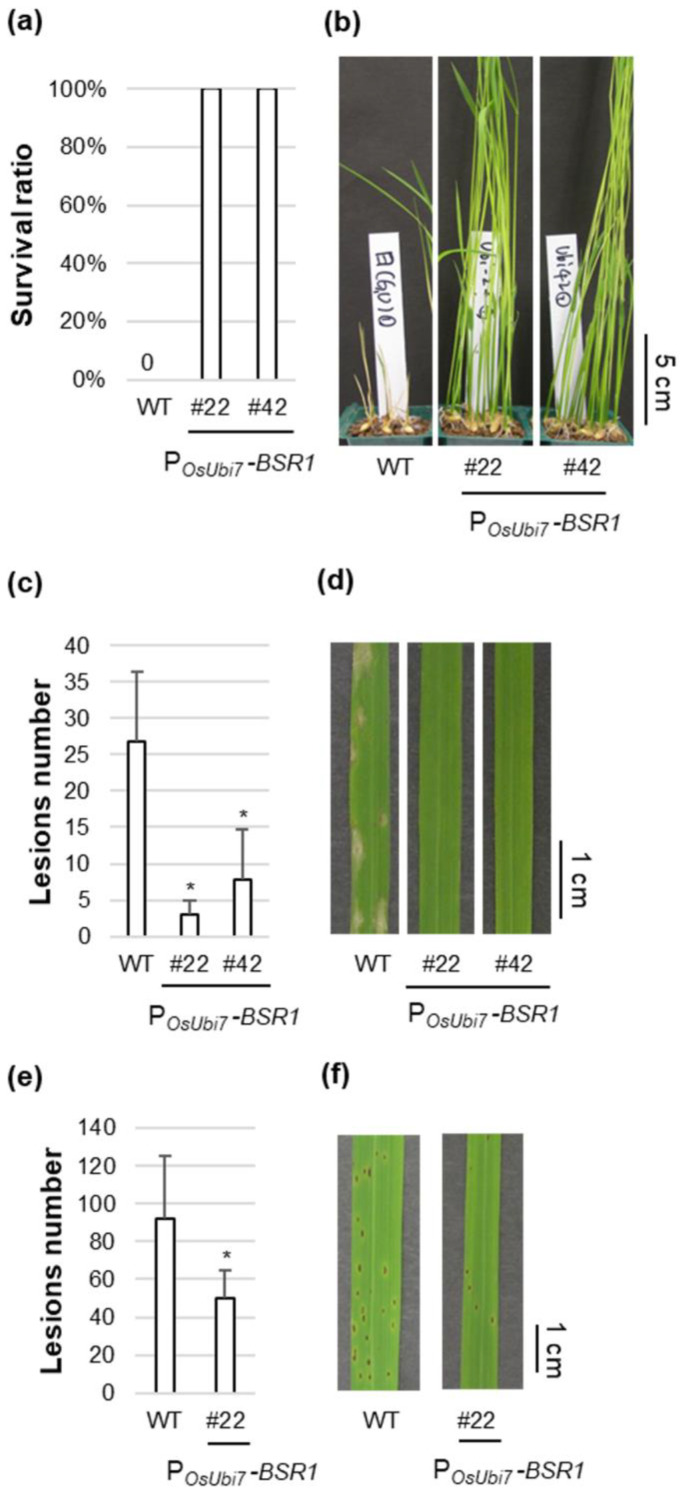
Disease resistance in the representative P*_OsUBi7_*-*BSR1* lines: (**a**,**b**) Disease resistance to *Burkholderia glumae* in P*_OsUbi7_*-*BSR1* lines. (**a**) Pre-germinated T4 seeds of P*_OsUbi7_*-*BSR1* and WT lines were inoculated with *B. glumae*. The inoculum concentration was OD_520_ = 0.0004. Survival ratio was calculated 8 d post-inoculation (*n* = 9–20). Tests were repeated three times with similar results. (**b**) Photographs of *B. gluma*-infected shoots in P*_OsUbi7_*-*BSR1* and WT lines 8 d post-inoculation. (**c**,**d**) Disease resistance to *Pyricularia oryzae* in P*_OsUbi7_*-*BSR1* lines. (**c**) Lesion numbers on *P. oryzae*-infected T4 leaves in P*_OsUbi7_*-*BSR1* and WT lines 6 d post-inoculation. The inoculum concentration was 1.3 × 10^5^ conidia/mL. Lesion numbers in P*_OsUbi7_*-*BSR1* plants were significantly lower than those in WT plants (* *p* < 0.05 by Dunnett’s test). Values are mean ± standard deviation (*n* = 3–7). (**d**) Photographs of leaves infected with *P. oryzae* in P*_OsUbi7_*-*BSR1* and WT 7 d post-inoculation. (**e**,**f**) Disease resistance to *Cochliobolus miyabeanus* in P*_OsUbi7_*-*BSR1* line. (**e**) Lesion numbers on *C. miyabeanus*-infected T4 leaves in P*_OsUbi7_*-*BSR1* and WT lines 4 d post-inoculation. The inoculum concentration was 1.4 × 10^4^ conidia/mL. Lesion numbers in P*_OsUbi7_*-*BSR1* plants were significantly lower than in WT plants (* *p* < 0.05 by *t*-test). Values are mean ± standard deviation (*n* = 6–7). (**f**) Photographs of leaves infected with *C. miyabeanus* in *P_OsUbi7_*-*BSR1* and WT lines 4 d post-inoculation.

The T4 generation of P*_OsUbi7_*-*BSR1* lines #22 and #42 was used as a representative for subsequent disease resistance tests. Overexpression of BSR1 was confirmed before disease resistance evaluation (Figure S3). First, the disease resistance to *B. glumae* was investigated. Although the WT plants succumbed to the infection, displaying 0% survival, lines #22 and #42 exhibited 100% survival, indicating resistance to bacterial seedling rot (Figure 4a,b). Subsequently, disease resistance to the fungus *P. oryzae*, which causes rice blast, was assessed. Lines #22 and #42 demonstrated robust resistance, with <1/8 and <1/3 of the lesions observed in comparison with the WT, respectively (Figure 4c,d). Subsequently, disease resistance to the fungus *C. miyabeanus*, which causes brown spots, was evaluated. Lines #22 and #42 showed strong resistance, with lesion numbers < 1/2 and <1/9, respectively, compared with the WT (Figure 4e,f and Figure S4). Overall, the P*_OsUbi7_*-*BSR1* lines (#22 and #42) maintained resistance to at least four pathogens, similar to the P*_ZmUbi_*-*BSR1* lines. Additionally, the descendants of lines #22 and #42 displayed resistance to panicle blast (Figure S5).

### 2.3. Resistance to Other Diseases in PPR1b-BSR1 Lines

**Figure 5:**
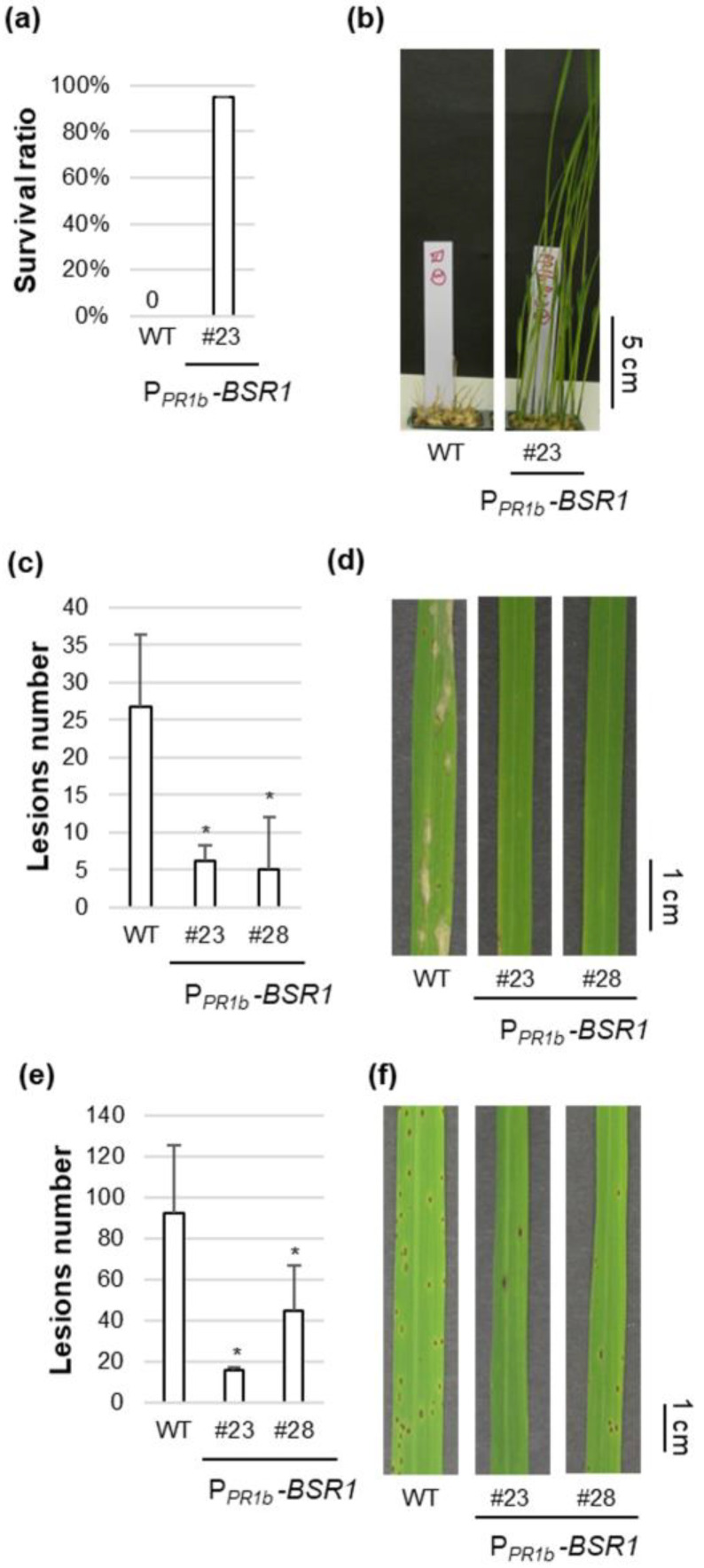
Disease resistance in the representative P*_PR1b_*-*BSR1* lines. (**a**,**b**) Disease resistance to *Burkholderia glumae* in P*_PR1b_*-*BSR1* line. (**a**) Pre-germinated T3 seeds of P*_PR1b_*-*BSR1* and WT lines were inoculated with *B. glumae*. The inoculum concentration was OD_520_ = 0.004. The survival ratio was calculated 7 d post-inoculation (*n* = 20). Tests were repeated three times with similar results. (**b**) Photographs of *B. glumae*-infected shoots in P*_PR1b_*-*BSR1* and WT 7 d post-inoculation. (**c**,**d**) Disease resistance to *Pyricularia oryzae* in P*_PR1b_*-*BSR1* lines. (**c**) Lesion numbers on *P. oryzae*-infected T1 leaves in P*_PR1b_*-*BSR1* and WT lines 6 d post-inoculation. The inoculum concentration was 1.3 × 10^5^ conidia/mL. Lesion numbers in P*_PR1b_*-*BSR1* plants were significantly lower than those in WT plants 6 d post-inoculation (* *p* < 0.05 by Dunnett’s test). Values are mean ± standard deviation (*n* = 4–7). (**d**) Photographs of leaves infected with *P. oryzae* in P*_PR1b_*-*BSR1* and WT 7 d post-inoculation. (**e**,**f**) Disease resistance to *Cochliobolus miyabeanus* in P*_PR1b_*-*BSR1* lines. (**e**) Lesion number on *C. miyabeanus*-infected T1 leaves in P*_PR1b_*-*BSR1* and WT lines 4 d post-inoculation. The inoculum concentration was 1.4 × 10^4^ conidia/mL. Lesion numbers in P*_PR1b_*-*BSR1* plants were significantly lower than in wild-type plants (* *p* < 0.05 by Dunnett’s test). Values are means ± standard deviation (*n* = 3–6). (**f**) Photographs of leaves infected with *C. miyabeanus* in P*_PR1b_*-*BSR1* and WT lines 4 d post-inoculation.

The progenies of P*_PR1b_*-*BSR1* lines #23 and #28 were used as representatives for disease resistance tests. First, disease resistance to *B. glumae* was investigated. The WT plants succumbed to the infection, displaying 0% survival. Lines #23 and #28 exhibited 95 and 35% survival, respectively, indicating their resistance to bacterial seedling rot (Figure 5a,b and Figure S6). Subsequently, the disease resistance to *P. oryzae* was evaluated. Lines #23 and #28 demonstrated strong resistance, with lesion numbers < 1/4 and <1/5, respectively, compared with the WT (Figure 5c,d). Third, disease resistance to *C. miyabeanus* was assessed. Lines #23 and #28 displayed strong resistance, with lesion numbers < 1/5 and <1/2 of that of the WT, respectively (Figure 5e,f). In summary, the P*_PR1b_*-*BSR1* lines (#23 and #28) maintained resistance to at least four pathogens, similar to the P*_ZmUbi_*-*BSR1* and P*_OsUbi7_*-*BSR1* lines.

### 2.4. Improvement of Undesirable Traits Detected in the Seeds of P_ZmUbi_-BSR1 Lines

**Figure 6:**
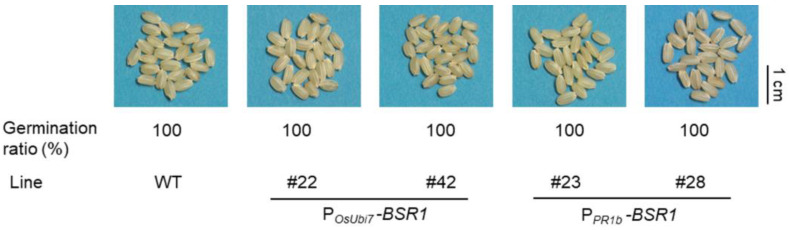
Improved seed phenotypes in the P*_OsUbi7_*-*BSR1* and P*_PR1b_*-*BSR1* lines. Photographs of dehusked grains from T4 seeds of the P*_OsUbi7_*-*BSR1* lines, T3 seeds of the P*_PR1b_*-*BSR1* lines, and WT. The grain color of these transgenic lines was white, as in the WT. The germination ratios (%) of these lines were similar to WT. *n* = 25.

As mentioned above, the two P*_OsUbi7_*-*BSR1* and two P*_PR1b_*-*BSR1* lines exhibited resistance to the four major diseases, mirroring the disease resistance observed in the P*_ZmUbi_*-*BSR1* lines. The grains of the T4 homo-seeds of the two P*_OsUbi7_*-*BSR1* lines and T3 homo-seeds of the two P*_PR1b_*-*BSR1* lines were white (Figure 6). Additionally, the issue of reduced germination in P*_ZmUbi_*-*BSR1* seeds was addressed, as these seeds demonstrated a 100% germination rate, similar to that of the WT (Figure 6). As described above, the successful utilization of *OsUbi7* and *OsPR1b* promoters preserved resistance to the four major diseases, effectively improved grain color, and resolved germination concerns in the seeds.

## 3. Discussion

When *BSR1* was expressed using the rice constitutive promoter P*_OsUbi7_* with moderate expression levels and the pathogen-inducible promotor P*_PR1b_* to adjust the expression level and timing in rice, both rice plants exhibited sufficient resistance to the four major diseases (rice blast, brown spot, bacterial leaf blight, and bacterial seedling rot), the P*_ZmUbi_*-*BSR1* rice plants did as well. This indicates that disease resistance can be conferred by appropriate expression of *BSR1* without using P*_ZmUbi_*. These promoters have been used to improve the growth of P*_ZmUbi_*-*WRKY45* rice plants with blast and bacterial leaf blight resistance [20,21]. In this study, we have shown that these promoters are useful for improving unfavorable phenotypes (such as the blackish color of brown rice and low germination rate) by overexpressing *BSR1*. Since the P*_OsUbi7_*-BSR1 and P*_PR1b_*-BSR1 lines are transgenic, they were grown in a small, isolated greenhouse, where exact yield comparisons are not feasible. Although no significant differences were observed compared to the WT, field evaluation is essential for accurate yield assessment.

*BSR1*-OX rice plants with P*_ZmUbi_*, which exhibit strong resistance to leaf blight and blast disease, show browning at the infection site [13,18]. In the present study, *BSR1*-expressing rice plants with P*_OsUBi7_* and P*_PR1b_* showed browning at the infection site, especially when infected with leaf blight, but this was not as remarkable as that in the case of P*_ZmUbi_*. We have previously reported that *BSR1* is involved in chitin-, peptidoglycan-, and lipopolysaccharide-triggered ROS production and defense responses and ROS production is enhanced in *BSR1*-OX rice [14,16]. Furthermore, it has been reported that *BSR1* is involved in plant responses to OsPeps and damage-associated molecular patterns (DAMPs), and these responses are enhanced in *BSR1*-OX rice [17]. Thus, the browning observed at the pathogen infection may be caused by cell death due to excessive ROS production by the recognition of microbe-associated molecular patterns (MAMPs) or DAMPs.

In P*_ZmUbi_*-*BSR1* rice plants, overexpression of *BSR1* caused a blackish grain color and reduced the seed germination rate, as described above (Figure 1). According to the Rice Expression Profile Database [22] (https://ricexpro.dna.affrc.go.jp/ (accessed on 11, March, 2024)), *BSR1* is transcribed at certain levels in leaves and roots at different growth stages and in developing panicles (young anthers, pistils, lemmas, palea, ovaries, embryos, and endosperms). Studies reported that ROS is involved in almost all the stages of growth, development, and differentiation and that they play important roles in programmed cell death and signal transduction [23,24,25]. Therefore, *BSR1*, which is expressed during ovary, embryo, and endosperm development after fertilization, may contribute to ROS generation in response to signals from ligands other than MAMPs and DAMPs in WT rice. Alternatively, it may respond to the MAMP signals produced by endophytes. Therefore, the overexpression of *BSR1* with P*_ZmUbi_* may induce the production of large amounts of ROS in the endosperm and embryo, causing partial darkening of the endosperm and cell death of the embryo, resulting in a reduced germination rate. In contrast, the improvement in brown rice color and germination rate in P*_OsUbi7_*-*BSR1* and P*_PR1b_*-*BSR1* rice seeds, similar to those in WT seeds, may be attributed to the moderate control of ROS production by adjusting *BSR1* expression to an appropriate level. The upstream signals of *BSR1* during panicle and seed development have not yet been elucidated; however, future studies could be conducted to uncover the ligands involved.

The *BSR1* gene has also been introduced into plants other than rice and has been shown to confer disease resistance. In *Arabidopsis*, the overexpression of *BSR1* under the *35S* promoter containing two enhancers resulted in disease resistance to three pathogens, including the bacterium *Pst* DC3000 as well as fungi *C. higginsianum* and *R. solani* [13,26]. When overexpressed in various plants under the same promoter as in *Arabidopsis*, *BSR1* confers disease resistance to *Pst* DC3000 and *R. solani* in tomatoes and to *R. solani* in torenia. *BSR1*-overexpressed sugarcane is resistant to one of the most serious diseases caused by the fungus *Sporisorium scitamineum* [26]. When *BSR1* was appropriately expressed in these plants, the transgenic plants exhibited normal growth and morphology similar to that of the WT and retained disease resistance, although a dwarf phenotype was observed in sugarcane with the highest expression level of *BSR1* [26]. By selecting appropriate promoters and fine-tuning the expression level of *BSR1*, it would be possible to generate plants with broad-spectrum disease resistance without compromising the growth in other crops as well as in rice.

Recently, P*_OsUbi7_*-*BSR1* rice was also reported to confer resistance to the chewing herbivore *Mythimna loreyi* Duponchel (Lepodoptera: Noctuidae), a rice pest [17]. As described above, by optimizing the expression level of *BSR1* using a suitable promoter such as *OsUbi7*, we were able to generate rice plants with BSR to four major diseases and one pest while minimizing the adverse effects of overexpression. The next step will be to determine whether these plants are effective against a wide range of pathogens and pests. Another question is whether similar traits can be maintained in a field. We cannot exclude the possibility that unfavorable phenotypes may occur due to somaclonal mutations derived from callus cultures during the generation process of transgenic rice when released into the field environment. We also cannot exclude the possibility of other problems arising from the accumulation of mutations over several generations. In such cases, it may be necessary to produce a larger number of transgenic rice plants and reduce the effects of cultural mutations through multiple backcrossings. Using a leaf-specific promoter may be effective because undesirable traits of *BSR1* were found in the seed.

## 4. Materials and Methods

### 4.1. Plasmid Construction and Transformation

The P*_PR1b_*-*BSR1* plasmid was constructed as follows: Briefly, the *WRKY45* cDNA of the *P_PR1b_*:*WRKY45*:*TT* plasmid [21] was replaced with *BSR1* cDNA. In detail, the *BSR1* cDNA (AK070024; Os09t0533600-01) provided by the Rice Genome Resource Center of the National Institute of Agrobiological Sciences was first amplified by PCR using the following primers: 5′-TTGATTAACTAAGCTTGTGCGTGCGTGCGTGCTTGC-3′ and 5′-TGATTTCAGCGGATCTCGTCTCTGTGTCTCTCTTT-3′. Subsequently, the *P_PR1b_*:*WRKY45*:*TT* plasmid was digested with *Bam*HI and partially digested with *Hin*dIII to excise the *WRKY45* cDNA from the vector, and the amplified *BSR1* cDNA was incorporated in place of the *WRKY45* cDNA using an In-Fusion HD Cloning Kit w/Cloning Enhancer (Takara Bio, Tokyo, Japan). Transgenic rice lines were obtained from Nipponbare using the resulting plasmid via the *Agrobacterium*-mediated method [27].

Construction of the P*_OsUbi7_*-*BSR1* plasmid and generation of P*_OsUbi7_*-*BSR1* rice lines have been previously reported briefly [17]. In detail, the *BSR1* cDNA was amplified by PCR using the following primers: 5′-GCAAAAGAAGAAGCTGTGCGTGCGTGCGTGCTTGC-3′ and 5′-TGATTTCAGCGGATCTGCTCTCTGTGTCTCTCTTT-3′. Subsequently, the *P _OsUbi7_*:*WRKY45*:*TT* plasmid was digested with *Bam*HI and partially digested with *Hin*dIII to excise the *WRKY45* cDNA from the vector. The *WRKY45* cDNA was then replaced with amplified *BSR1* cDNA using an In-Fusion HD Cloning Kit with Cloning Enhancer.

### 4.2. RNA Extraction and Quantitative Real-Time (qRT)-PCR Analysis

Total RNA was extracted and purified from rice leaves using the Sepasol-RNA Super G reagent (Nacalai Tesque, Kyoto, Japan) according to the manufacturer’s protocol. First-strand cDNAs were synthesized from equal amounts of total RNA in a volume of 5 μL using the PrimeScript RT Reagent Kit (Takara, Tokyo, Japan), according to the manufacturer’s protocol. The Thermal Cycler Dice TP800 system (Takara) and Kapa SYBR FAST qPCR kit (Kapa Biosystems, Cape Town, South Africa) were used for qRT-PCR analysis according to the manufacturer’s instructions. Primers used for qRT-PCR were as follows: for *BSR1*, 5′-CCGGGACTTCAAAGCATCTAAC-3′ and 5′-TGTTGGTCCCTCCCTTGCT-3′; for *Rubq1*, 5′-GGAGCTGCTGCTGTTCTAGG-3′ and 5′-TTCAGACACCATCAAACCAGA-3′, serving as an internal control. *BSR1* transcript levels were normalized to the endogenous rice reference gene *Rubq1*. The relative expression level of each gene was calculated using the 2^−ΔΔCt^ expression ratio, which corrects for gene-specific PCR amplification efficiencies [28].

Overexpression of *BSR1* cDNA was confirmed by qRT-PCR analysis of independent T0 plants from the P*_OsUbi7_-BSR1* lines, and their progenies were subsequently utilized in further experiments.

### 4.3. Plant Materials and Culture

Rice (*Oryza sativa* L. cv. Nipponbare) was used as the WT control. Seeds from T1 to T4 of the P*_OsUbi7_*-*BSR1* and P*_PR1b_*-*BSR1* lines were sown on half-strength MS medium (Wako, Osaka, Japan) containing 3% sucrose, 0.4% Gelrite (Wako), and hygromycin B (30 μg/mL; Wako), and the hygromycin-resistant seedlings were selected on this medium. WT seeds were sown and grown on the same medium without hygromycin B. WT and hygromycin-resistant transgenic seedlings were then transplanted into pots containing soil (Bonsol no. 2; Sumitomo Kagaku Kougyo, Osaka, Japan) and grown in a greenhouse at 27–30 °C, as previously described [19].

### 4.4. Pathogens and Pathogen Cultures

The bacterial isolates used in this study were T7174 (MAFF311018, race I) and T7133 (MAFF311020, race III) from *Xanthomonas oryzae* pv. *oryzae* (*Xoo*), and AZ8204 (MAFF301682) from *Burkholderia glumae*. Additionally, the fungal isolates were Kyu89-246 (MAFF101506, race 003.0) from *Pyricularia oryzae* and H11-42-1 from *Cochliobolus miyabeanus*. Culture procedures for these pathogens were conducted as previously described [18].

### 4.5. Bacterial and Fungal Pathogen Resistance Assay

For the *Xoo* resistance tests, a suspension of *Xoo* adjusted to OD_600_ = 0.3 in water was inoculated onto seedlings of WT and transgenic lines at the 5- to 10-leaf stage using the clip and dip method, as previously described [18]. For *B. glumae* resistance assays, pre-germinated seeds of WT and transgenic lines were soaked in a suspension of *B. glumae* adjusted to OD_520_ = 0.0004–0.004 and subjected to vacuum treatment following previously described protocols [18]. For *P. oryzae* resistance assays, conidia of a compatible race 003 (isolate Kyu89-246) were suspended in 0.01% Tween 20 at a density of 1.3 × 10^5^/mL and sprayed onto seedlings of WT and transgenic lines at the 5-leaf stage, as previously described [18]. For *C. miyabeanus* resistance assays, a suspension of *C. miyabeanus* conidia adjusted to 1.4–5 × 10^4^/mL was sprayed onto seedlings of WT and transgenic lines at the 7-leaf stage, following previously established procedures [18].

### 4.6. Brown Rice Color Evaluation and Germination Test

The brown rice color of WT and transgenic rice seeds (25 seeds each) was visually evaluated. For the germination test, sterilized brown rice was sown in half-strength MS medium without hygromycin B. After 3 d, seed germination was examined, and the germination ratio was calculated.

## Data Availability

Data are contained within the article.

## Supplementary Materials

The following supporting information can be downloaded at https://www.mdpi.com/article/10.3390/plants13081138/s1, Figure S1: Disease resistance to *Xanthomonas oryzae* pv. *oryzae* (T7174, race I) in P*_OsUbi7_*-*BSR1* T1 lines; Figure S2: Disease resistance to *Xanthomonas oryzae* pv. *oryzae* (T7133, race III) in P*_OsUbi7_*-*BSR1* T1 lines; Figure S3: *BSR1* expression levels in P*_OsUbi7_*-*BSR1* T4 lines; Figure S4: Disease resistance to *Cochliobolus miyabeanus* in P*_OsUbi7_*-*BSR1* T4 line; Figure S5: Disease resistance to panicle blast caused by *Pyricularia oryzae* (isolate Kyu89-246) in the descendants of the P*_OsUbi7_*-*BSR1* #22 and #42 lines; Figure S6: Disease resistance to *Burkholderia glumae* in P*_PR1b_*-*BSR1* T2 lines; Method S1: Evaluation of panicle blast resistance [29,30,31].
