# Supplementary material for: Improvement of Broad-Spectrum Disease-Resistant Rice by the Overexpression of BSR1 via a Moderate-Strength Constitutive Promoter and a Pathogen-Inducible Promoter
Source: Plants (Basel). 2024 Apr 18;13(8):1138. doi: 10.3390/plants13081138 (PMC11054640; doi:10.3390/plants13081138)
Supplement: Supplementary file 1 [file plants-13-01138-s001.zip › plants-2946972-supplementary.pdf]

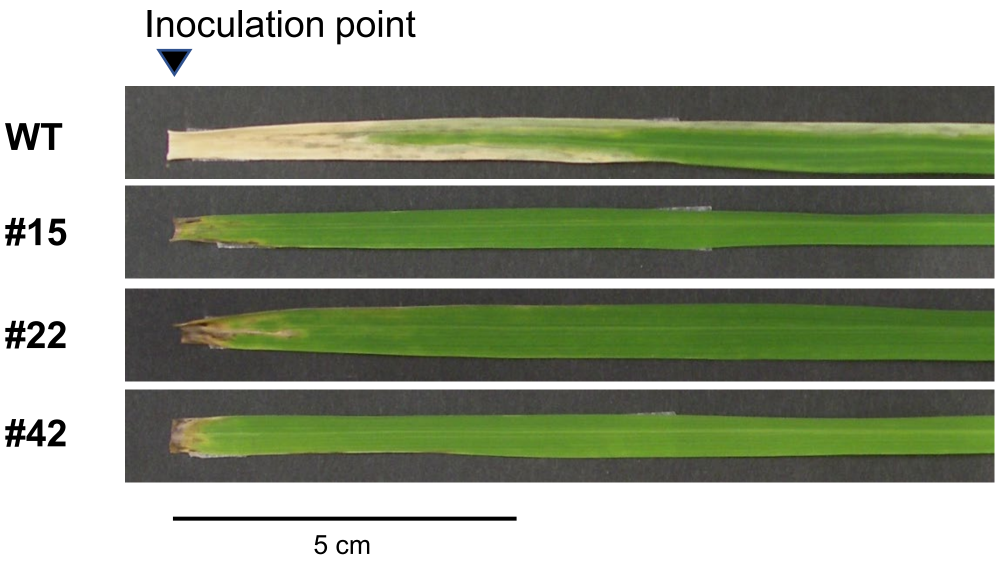

Figure S1. Disease resistance to *Xanthomonas oryzae* pv. *oryzae* (T7174, race I) in P$_{OsUbi7}$-*BSR1* T1 lines. Top leaves (L6-L8) of P$_{OsUbi7}$-*BSR1* and WT lines were inoculated *with X. oryzae* pv. *oryzae* (T7174). The arrowhead indicates the point of inoculation.

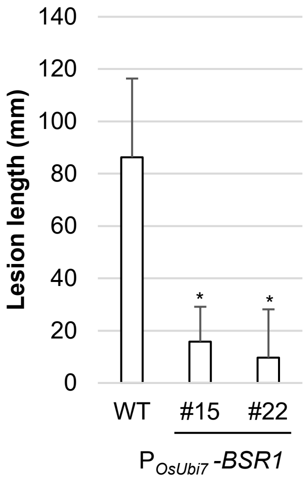

Figure S2. Disease resistance to *Xanthomonas oryzae* pv. *oryzae* (T7133, race III) in P_{OsUbi7}-*BSR1* T1 lines. Top leaves (L6-L8) of P_{OsUbi7}-*BSR1* and WT plants were inoculated *with X. oryzae* pv. *oryzae* (T7133). Lesion lengths in P_{OsUbi7}-*BSR1* lines were significantly lower than those in wild-type (WT) plants (*$P < 0.05$ by Dunnett's test). Values are mean ± SD (n = 6-18).

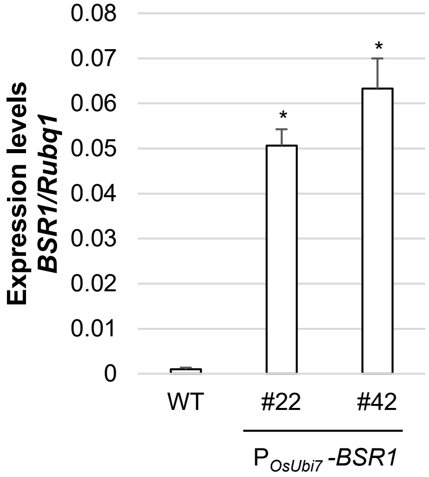

Figure S3. ***BSR1*** **expression levels in P$_{\boldsymbol{OsUbi7}}$-*BSR1*** **T4 lines.** *BSR1* expression levels in P$_{OsUbi7}$-*BSR1* lines were significantly higher than those in wild-type (WT) plants (**P* < 0.05 by Dunnett's test). Values are mean ± SD (n = 3).

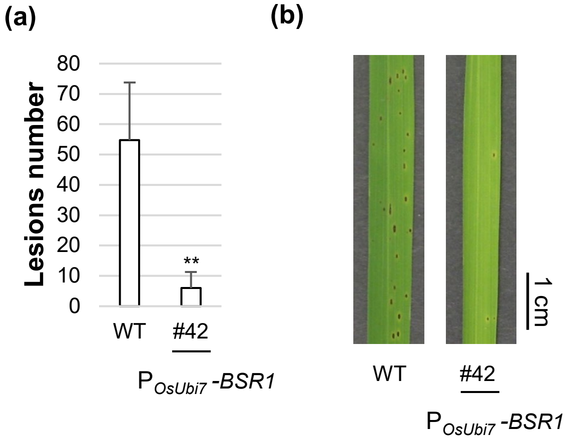

Figure S4. Disease resistance to *Cochliobolus miyabeanus* in P_{OsUbi7}-*BSR1* T4 line. (a) Lesion numbers on *C. miyabeanus* infected T4 leaves in P_{OsUbi7}-*BSR1* and WT lines 4 d after inoculation. The inoculum concentration was 5×10^4 conidia/ ml. Lesion numbers in P_{OsUbi7}-*BSR1* plants were significantly lower than in WT plants 4 d after inoculation (**$P < 0.01$ by t-test). Values are mean ± SD (n = 6-7). (b) Photographs of leaves infected with *C. miyabeanus* in P_{OsUbi7}-*BSR1* and WT lines 4 d after inoculation.

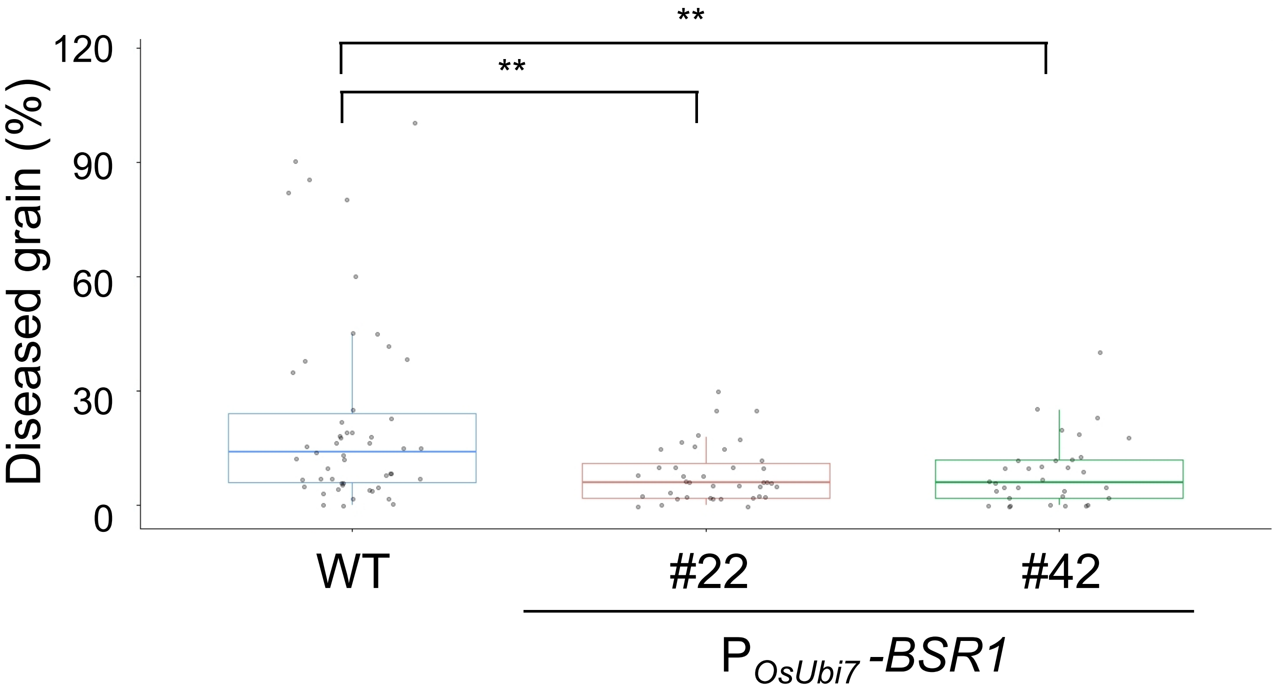

Figure S5. Disease resistance to panicle blast caused by *Pyricularia oryzae* (isolate Kyu89-246) in the descendants of the P$_{OsUbi7}$-*BSR1* #22 and #42 lines. Kruskal-Wallis test with Steel's post-hoc test was used to analyze significant differences between these group (**P<0.01).

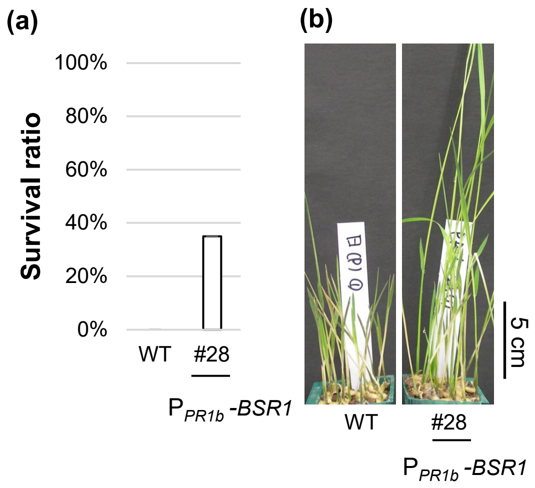

Figure S6. Disease resistance to *Burkholderia glumae* in P_{PR1b}-*BSR1* T2 lines. (a) Pre-germinated T2 seeds of P_{PR1b}-*BSR1* and wild-type (WT) lines were inoculated with *B. glumae*. The inoculum concentration was OD_{520} = 0.0004. Survival ratio was calculated 8 d after inoculation (n = 20). Tests were repeated thrice with similar results. (b) Photographs of *B. glumae* infected shoots in P_{PR1b}-*BSR1* and WT lines 8 d after inoculation.

Method S1. *Evaluation of panicle blast resistance*

The young seedlings were transplanted into plastic pots, grown in the glasshouse room of the NARO, and used for inoculation within 10 d from the day when the neck of the panicle emerged. Spores of race 003.0, a blast fungus pathogenic to WT and P_{OsUbi7} -*BSR1* lines, were suspended in 0.01% Tween 20 and sprayed with 20 mL per pot at a concentration of 1.0×10^5 conidia/ mL; and the plants were kept in a dew chamber for 20 h at 24.5 °C and grown in a glasshouse. The proportions of diseased grains per panicle were examined approximately 3 weeks after inoculation [1, 2]. Statistical analyses were conducted using EZR (Saitama Medical Center, Jichi Medical University, Saitama, Japan), which is a graphical user interface for R [3]. The Kruskal–Wallis test with Steel's post-hoc test was used to analyze significant differences between these group (**$P < 0.01$).

References

1. Hayashi, N.; Inoue, H.; Kato, T.; Funao, T.; Shirota, M.; Shimizu, T.; Kanamori, H.; Yamane, H.; Hayano-Saito, Y.; Matsumoto, T.; Yano, M.; Takatsuji, H., Durable panicle blast-resistance gene Pb1 encodes an atypical CC-NBS-LRR protein and was generated by acquiring a promoter through local genome duplication. *Plant J.* **2010,** 64, (3), 498-510.
2. Inoue, H.; Hayashi, N., The Panicle Blast Resistance Mechanism of in the Rice Cultivar Miyazaki-mochi is Independent from that of. *Jarq-Jpn Agr Res Q* **2019,** 53, (4), 289-293.
3. Kanda, Y., Investigation of the freely available easy-to-use software 'EZR' for medical statistics. *Bone Marrow Transplant.* **2013,** 48, (3), 452-458.
